# Should essays and other “open-ended”-type questions retain a place in written summative assessment in clinical medicine?

**DOI:** 10.1186/s12909-014-0249-2

**Published:** 2014-11-28

**Authors:** Richard J Hift

**Affiliations:** Clinical and Professional Practice Research Group, School of Clinical Medicine, University of KwaZulu-Natal, Durban, 4013 South Africa

**Keywords:** Assessment, Conceptual change, Essay, Mental models, MEQ, Multiple choice

## Abstract

**Background:**

Written assessments fall into two classes: *constructed-response* or *open-ended* questions, such as the essay and a number of variants of the short-answer question, and *selected-response* or *closed-ended* questions; typically in the form of multiple-choice. It is widely believed that constructed response written questions test higher order cognitive processes in a manner that multiple-choice questions cannot, and consequently have higher validity.

**Discussion:**

An extensive review of the literature suggests that in summative assessment neither premise is evidence-based. Well-structured open-ended and multiple-choice questions appear equivalent in their ability to assess higher cognitive functions, and performance in multiple-choice assessments may correlate more highly than the open-ended format with competence demonstrated in clinical practice following graduation. Studies of construct validity suggest that both formats measure essentially the same dimension, at least in mathematics, the physical sciences, biology and medicine. The persistence of the open-ended format in summative assessment may be due to the intuitive appeal of the belief that synthesising an answer to an open-ended question must be both more cognitively taxing and similar to actual experience than is selecting a correct response. I suggest that cognitive-constructivist learning theory would predict that a well-constructed context-rich multiple-choice item represents a complex problem-solving exercise which activates a sequence of cognitive processes which closely parallel those required in clinical practice, hence explaining the high validity of the multiple-choice format.

**Summary:**

The evidence does not support the proposition that the open-ended assessment format is superior to the multiple-choice format, at least in exit-level summative assessment, in terms of either its ability to test higher-order cognitive functioning or its validity. This is explicable using a theory of mental models, which might predict that the multiple-choice format will have higher validity, a statement for which some empiric support exists. Given the superior reliability and cost-effectiveness of the multiple-choice format consideration should be given to phasing out open-ended format questions in summative assessment. Whether the same applies to non-exit-level assessment and formative assessment is a question which remains to be answered; particularly in terms of the educational effect of testing, an area which deserves intensive study.

**Electronic supplementary material:**

The online version of this article (doi:10.1186/s12909-014-0249-2) contains supplementary material, which is available to authorized users.

## Background

### Learning and the stimulation of learning by assessment

Modern definitions of learning, such as that attributed to Siemens: “Learning is a continual process in which knowledge is transformed into something of meaning through connections between sources of information and the formation of useful patterns, which generally results in something that can be acted upon appropriately, in a contextually aware manner” [1],[2] essentially stress two points: firstly, that learning requires a much deeper, effortful and purposeful engagement with the material to be learned than the acquisition of factual knowledge alone; secondly, that learned knowledge does not exist in a vacuum; its existence is inferred from a change in the learner’s behaviour. This has led transfer theorists to postulate that knowledge transfer is the basis of *all* learning, since learning can only be recognised by observing the learner's ability to display that learning later [3],[4].

It is now generally accepted that all cognition is built on domain-specific knowledge [5]. Content-light learning does not support the ability to transfer knowledge to new situations and a comprehensive store of declarative or factual knowledge appears essential for transfer [4]. Furthermore, a high order of understanding and contextualization must accompany the declarative knowledge if it is to be successfully applied later. Where transfer – in other words, the successful application of knowledge to new situations – has been shown, the common factor appears to be deep learning, and the abstraction of general principles [6]-[8].

**Figure 1:**
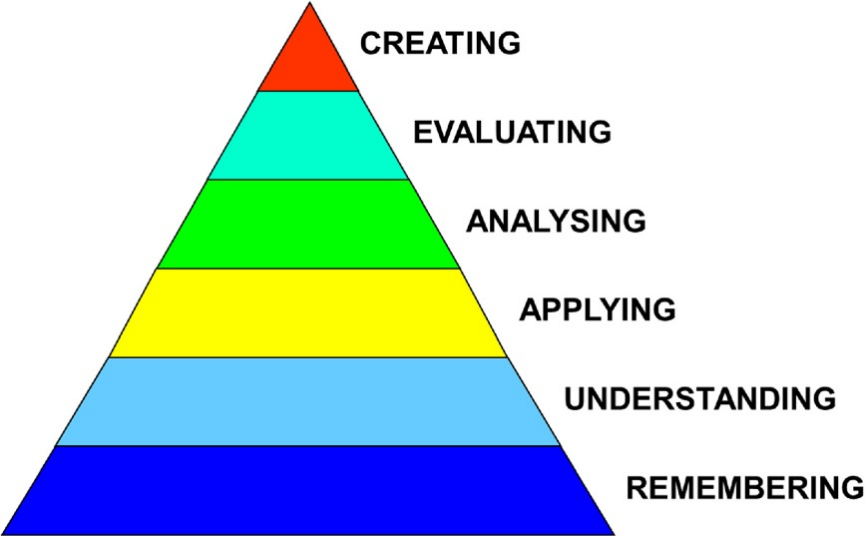
**Modified bloom’s taxonomy [** [11]**].**

**Figure 2:**
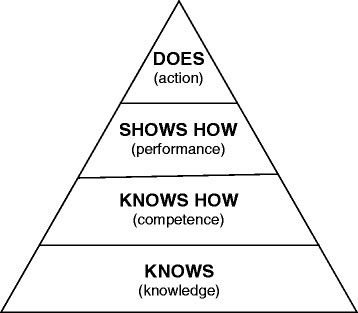
**Miller’s pyramid of assessment of clinical skills, competence and performance [** [15]**].**

Indeed, knowledge may be acquired and held at varying depths. Aspects of this are reflected in the cognitive levels of learning constituting Bloom's taxonomy of learning [9]-[14] (Figure 1); the varying levels of clinical competence and performance described in Miller’s pyramid [15] (Figure 2) and the stages of proficiency postulated by Dreyfus and Dreyfus [16]. The extent to which different assessment formats measure proficiency over the entire range of complexity of understanding and performance is one of the central issues in assessment.

Assessment is central to the educational process, and has benefits beyond that of measuring knowledge and competence alone; principally in directing and stimulating learning, and in providing feedback to teachers and learners [17]. Recent research supports a critical role for assessment in consolidating learning, and strengthening and facilitating memorisation and recall. There is accumulating evidence that the process of stimulating recall through testing enhances learning and retention of learned material. This has been termed the *testing effect*, and several hypotheses have been put forward to explain it, including increased cognitive effort, conceptual and semantic processing, and increased attention to the properties distinguishing the learnt item from similar items, which strengthens the relationship between the cue which triggers the memory and the memory item itself [18],[19]. It appears to be principally the act of retrieving information from memory which strengthens knowledge and knowledge retention [20],[21], irrespective of whether retrievable is covert or overt [22]. Importantly, high-level questions appear to stimulate deeper conceptual learning and better learning retention then those pitched at a lower level [23]. A number of strategies have been proposed to exploit this in educational practice, including those recently summarised for use in medical education [24]. This is in a sense related to the “generation effect”, where it has been shown that spontaneously generating information as opposed to learning it passively improves subsequent recall [18],[19].

### Assessment in educational practice

It is accepted that standards of assessment are inherently variable. There is therefore an obligation, in summative assessment, to ensure that assessment meets certain minimum criteria [25]. Achieving this in the individual instance is challenging, given the wide range of skills and knowledge to be assessed, marked variation in the knowledge of assessment of those who must assess and the highly variable environments in which the assessment takes place. There is now an extensive literature on assessment, in terms of research, guidelines and recommendations [26],[27]. Importantly, modern approaches recognise that no single form of assessment is suitable for every purpose, and stressed the need for *programmatic assessment*, which explicitly recognises that assessment is best served by a careful combination of a range of instruments matched to a particular purpose at each stage of the learning cycle, such as for formative, diagnostic or summative purposes [25],[26],[28].

### Written assessment

Despite the proliferation of assessment methodologies which attempt to test the competence of medical students directly, such as OSCE, OSPE, case-based assessment, mini-CEX and workplace-based assessment, written assessments remain in widespread use. Much of the knowledge base required by the clinician is not necessarily testable in the performance format. Additionally, in comparison with most practical assessment formats, written tests are easier to organize and deliver, requiring little more than pen and paper or a computer, a venue, question setters and markers who need not be physically present.

In general, all forms of written assessment may be placed into one of two categories. *Constructed response* or *open-ended* questions include a variety of written formats in which the student is required to *generate* an answer spontaneously in response to a question. The prototypical example is the essay. There are many variants including short answer questions (SAQ), mini-essay questions, single-word and single-sentence questions and the modified essay question (MEQ). The *selected-response* or *closed-ended* format is typified by the multiple-choice question (MCQ) assessment, where candidates *select* the most appropriate answer from a list of options rather than generating an answer spontaneously. Many variants of the multiple-choice format have been used: current best practice recommends the use of *one-best-answer* (of three, four or five possible answers), and *extended matching item* (EMI) formats [29]. In this debate I shall use the term *open-ended* when referring to the constructed-response format, and *multiple-choice* as a synonym for the selected-response format.

All high-stakes assessments should meet an adequate standard in terms of quality and fairness, as measured by a number of parameters, summarised recently in a consensus statement [30]. Principal among these are the classic psychometric parameters of reproducibility (reliability or consistency; that a result would not essentially change with retesting under similar conditions), and validity or coherence, which I describe in detail below. Other important measures by which assessments should be judged are equivalence (assessments administered at different institutions or during different testing cycles produce comparable outcomes), feasibility (particularly in terms of efficiency and cost effectiveness), educational effect (the student who takes the assessment is thereby motivated to undertake appropriate learning), catalytic effect (the assessment provides outcomes that, when fed back into the educational programme, result in better teaching and learning) and acceptability to both teachers and learners.

It is generally accepted that the multiple-choice format, in contrast to the open-ended format, has high reliability and is efficient, a consequence primarily of wide sampling, and to a lesser extent, of its objectivity. In support of the open-ended format, it has been widely held that this format is superior at testing higher cognitive levels of knowledge and has greater validity. This belief is intuitively appealing and appears to represent the viewpoint of many of those involved in medical assessment, including those with extensive knowledge and experience in medical education. In an attempt to gain the best of both formats, there has been a shift from the prototypical essay towards newer formats comprising a larger number of short, structured questions, a development intended to retain the perceived benefit of the open-ended question with the superior reliability of the MCQ.

Thus the two formats are generally seen to be in tension, MCQ being significantly more reliable, the open-ended format having greater validity. In this debate I will compare the performance of the open-ended format with MCQ in summative assessment, particularly in final exit examinations. I draw attention to the large body of evidence which supports the view that, in summative assessment, the multiple-choice format is intrinsically able to provide all the value of the open-ended format and does so more reliably and cost effectively, thus throwing into question the justification for the inclusion of the open-ended format in summative assessment. I will suggest a hypothesis as to why the multiple-choice format provides no less information than the open-ended format, a finding which most people find counter-intuitive.

A critical concept is that assessment is not only *of* learning, but also *for* learning [27],[31]. In the first case, the purpose of assessment is to determine whether that which is required to be learnt has in fact been learnt. In the second case, it is acknowledged that assessment may in itself be a powerful driver for learning at the cognitive level. This is supported by a body of evidence indicating the powerful effect of assessment on strengthening memorisation and recall [20],[22],[23]. In this debate I concentrate primarily on summative assessment in its role as *assessment of learning*; one must however remain aware that those methods of assessment best suited to such summative assessment may not be identical to those best suited to *assessment for learning*; indeed, it would be surprising if they were.

For the first part of the 20^th^ century, written assessment in medicine consisted largely of essay-writing [30]. Multiple-choice assessment was developed for psychological testing by Robert Yerkes immediately before the First World War and then rapidly expanded for the testing of army recruits. Yerkes was interested in assessing learning capacity—not necessarily human—and applied it to crows [32] and pigs [33] as well as psychiatric patients and mentally challenged subjects, a group among whom it was widely used for a number of years thereafter [34],[35]. Application to educational assessment has been credited to Frederick J. Kelly in 1914, who was drawn to it by its efficiency and objectivity [36].

Throughout its history, the multiple-choice format has had many detractors. Their principal arguments are that closed-ended questions do not stimulate or test complex constructive cognitive processes, and that if the ability to construct rather than choose a correct answer is not actively assessed, there is a potential that it will be neither taught nor learnt [37]-[41].

As Rotfield has stated: "Students proudly show off their high grades, from multiple-choice exams, as if their future careers will depend on knowing which choice to make instead of discerning which choices exist" [42]. Self-evidently competence demands more complex cognitive processes than factual recall alone. The ability to invoke these higher levels of cognition is clearly a skill which should be explicitly assessed. Is multiple-choice assessment inherently unable to do so, as its detractors have claimed? The belief that open-ended questions test high-order cognitive skills whereas multiple-choice questions do not and that therefore by inference open-ended questions evoke and test a reasoning process which is more representative of real-life problem-solving than multiple-choice, is a serious concern which I address in this review. We begin however with a comparison of the two formats in terms of reproducibility and feasibility.

## Discussion

### Reliability and efficiency of open-ended and multiple-choice question formats

Wider sampling greatly increases reproducibility, compensating as it does for unevenness in a candidate’s knowledge, varying quality of questions and even the personality of examiners [43],[44]. That the reproducibility of the multiple-choice format is much higher than that of the open-ended format is borne out in numerous studies comparing the two formats [45]-[47]. Recognition of these shortcomings has led to the design of open-ended-formats specifically intended to increase reproducibility and objectivity, while maintaining the supposed advantages of this format in terms of validity. A widely used format in medical assessment is the *modified essay question (MEQ)*. The format is of a clinical scenario followed by a series of sequential questions requiring short answers. This was expressly designed to bridge a perceived gap between multiple-choice and SAQ as it was believed that it would prove better at testing high-order cognitive skills than multiple-choice while allowing for more standardised marking than the standard open-ended question [45].

Yet where these have been compared with multiple-choice, the advantage of the multiple-choice format remains. A large number of questions and multiple markers are required in order to provide acceptable reliability for MEQs and essay questions [45]. Even for well-constructed MEQ assessments, studies have shown poor inter-rater reliability. Thus in an MEQ paper in a final undergraduate medical exit examination marked in parallel by several assessors, statistically significant differences between the scores of the different examiners were shown in 50% of the questions, as well as significant differences in the median scores for the examination as a whole [47]. Nor were these differences trivial; a substantial difference in outcome in terms of likelihood of failure were shown. This is cause for concern. Schuwirth *et al*. have stressed the necessity for interpreting reliability in terms of outcome, particularly in terms of pass/fail misclassification, and not merely in terms of numeric scores such as Cronbach’s alpha [27]. In this and other such studies the open-ended questions were of the highest possible quality practically achievable, typically MEQ's carefully prepared by skilled question writers working in teams, reviewed for appropriateness and scored using an analytic scoring scheme designed to minimise inter-rater variability. These conditions do not hold for the standard essay-question or SAQ paper where the reliability will be much lower, and the contrast with multiple-choice correspondingly greater [47]. Open-ended items scored on a continuum, such as 0-100%, have much lower inter-rater reliability than those scored against a rigid marking schedule. Therefore the discrepancy in reliability for the "graded essay" marked on a continuum versus multiple-choice is much larger than it is for more objectively scored open-ended formats.

In contrast to the open-ended question format, the multiple-choice is objective and allows multiple sampling of a subject. The result is high reproducibility. Furthermore it substantially reduces the potential for a perception of examiner bias, and thus the opportunity for legal challenge by the unsuccessful candidate [48]. The multiple-choice format is efficient. Lukhele *et al*. studied a number of national university-entrance examinations which included both multiple-choice items and essay questions [49]. They found that 4-8 multiple-choice items provided the same amount of information as a single essay, and that the essay’s efficiency in providing information about the candidate’s ability per minute of testing was less than 10% of that of an average multiple-choice item. For a middle-level examinee, approximately 20 times more examination time was required for an essay to obtain the same information as could be obtained from a multiple-choice assessment. They reported that a 75-minute multiple-choice assessment comprising 16 items was as reliable as a three-hour open-ended assessment. Though the relative gain in efficiency using multiple-choice in preference to essay questions varies according to subject, it is an invariable finding [49].

Though the initial development of an multiple-choice assessment is labour-intensive, this decreases with increasing experience on the part of item-writers, and decreases further once a question bank has been developed from which questions can be drawn for re-use. The lower efficiency of the open-ended question is not restricted to examination time but also the requirement for grading by examiners. Typically an open-ended test requires from 4 to 40 times as long to administer as a multiple-choice test of equivalent reliability [50]. In one study, the cost of marking the open-ended items was 300 times that of the multiple-choice items [49]; the relative cost of scoring the papers may exceed a factor of 1000 for a large examination [50].

The multiple-choice format thus has a clear advantage over open-ended formats in terms of reproducibility, efficiency and cost-effectiveness. Why then are open-ended questions still widely used? Principally this is because of a belief that essay-type questions, SAQ and their variants test higher-order cognitive thinking in a manner that MCQ cannot, and consequently have higher validity. It has been repeatedly stated that the MCQ format is limited in its ability to test deep learning, and is suitable for assessing facts only, whereas open-ended questions assess dynamic cognitive processes such as the strength of interconnected rules, the use of the mental models, and the mental representations which follow [37]-[39]; in short that open-ended questions permit the assessment of logical and reasoning skills in a manner that multiple-choice does not [40],[41]. Is there evidence to support these assertions?

### The ability to test higher-order cognitive skills

The revised Bloom's taxonomy of learning [9]-[12] is helpful in evaluating the level of cognition drawn upon by an assessment (Figure 1). By convention, assessment questions targeting the first two levels, are regarded as low-level questions, the third level as intermediate, and the fourth to sixth levels as high-level.

Those who understand the principles underlying the setting of high-quality multiple-choice items have no difficulty in accepting that multiple-choice is capable of assessing high-order cognition [10],[13],[14]. The shift from true-false questions, (which in order to avoid ambiguity frequently test factual information only) to the one-best-answer and EMI formats have facilitated this [29]. Indeed, there exist well-validated instruments specifically designed to assess critical thinking skills and to measure their development with progress through college-level educational programs, which are entirely multiple-choice based, such as the California Critical Thinking Skills Test [51],[52]. Schuwirth and Van der Vleuten [48] make a distinction between *context-rich* and *context-free* questions. In clinical assessment, a context-rich question is typically presented as a case vignette. Information within the vignette is presented to candidates in its original raw format, and they must then analyse, interpret and evaluate this information in order to provide the answer. The stimulus reflects the question which the candidate must answer and is therefore relevant to the content of the question. An example of a final-year question in Internal Medicine is shown in the following example. Such a question requires analysis (*What is the underlying problem?*), application (*How do I apply what I know to the treatment of this patient?*) and evaluation (*Which of several possible treatments is the most appropriate?*), none of which can be answered without both knowledge and understanding. Thus 5 of Bloom’s 6 levels have been tested.

**Example of a context-rich multiple-choice item in internal medicine**

*A24-year-oldwoman is admitted to a local hospital with a short history of epistaxis. On examination she is found to have a temperature of 36.9°C. Sheis wasted, has significant generalised lymphadenopathy and mild oral candidiasis but no dysphagia. A diffuse skin rash is noticed, characterised by numerous small purple punctate lesions.A full blood count shows a haemoglobin value of 110 g/L, a white cell count of 3.8×10*^*9*^*per litre and platelet count of 8.3×10*^*9*^*per litre. Which therapeutic intervention is most urgently indicated in this patient?*

- *Antiretroviral therapy*
- *Fluconazole*
- *Imipenem*
- *Prednisone*
- *Platelet concentrate infusion*

None of the options offered are obviously unreasonable or easily excluded by the candidate who attempts to shortcut the cognitive processes required in answering it by searching for clues in the options themselves. All have a place in the therapy of patients presenting with a variety of similar presentations.

Answering this item requires:

**Analysis**. In order to answer this item successfully, the candidate will have to recognise (1) that this patient is highly likely to be HIV-positive (given the lymphadenopathy, evidence of oral candidiasis and the high local prevalence of HIV), (2) that the presentation is suggestive of immune thrombocytopenic purpura (given the epistaxis, skin manifestations and very low platelet count), (3) that other commonly-seen concomitant features such as severe bacterial infection and extensive esophageal candidiasis are excluded by a number of negative findings.

**Evaluation**. Further, in order to answer this item successfully, the candidate will have to (1) consider the differential diagnosis for the principal components of the clinical vignette and, by process of evaluation, decide which are the most likely; (2) decide which of the diagnoses require treatment most urgently, (3) decide which form of therapy will be most appropriate for this.

**Knowledge, understanding and application**. It is utterly impossible to “recognise” the correct answer to this item without having worked through this process of analysis and evaluation, and the knowledge required to answer it must clearly be informed by deep learning, understanding and application. Hence five of the six levels of Bloom’s taxonomy have been tested. Furthermore it would appear an eminently reasonable proposition that the candidate who correctly answers this question will indeed be able to manage such a patient in practice, hence implying structural validity.

Though guessing has a 20% chance of providing the correct answer, this will be eliminated as a factor by assessing performance across multiple such items and applying negative marking to incorrect answers.

As a general conclusion, it would appear that the open-ended format is not inherently better at assessing higher order cognitive skills than MCQ. The fundamental determinant is the way in which the question is phrased in order to stimulate higher order thinking; if phrased inappropriately, the open-ended format will not perform any better than MCQ. A crucial corollary is that in comparing formats, it is essential to ensure that MCQ questions crafted to elicit high order thinking (particularly those which are context-rich) are compared with open-ended questions crafted to the same level; it is inappropriate to compare high-order items in one format with low order items in the other. Several studies have investigated the effect of the stimulus on thought processes in the open questions and have shown that the *stimulus format is more important than the response format*. Scores on questions in open-ended format and multiple-choice format correlate highly (approaching 100%) for context-rich questions testing the same material. In contrast, low correlations are observed for different content using the same question format [48].

In response to the low objectivity and reliability of the classic essay-type questions, modified open-ended formats have evolved which typically combine short answers, carefully crafted questions and rigid marking templates. Yet this increase in reliability appears to come at a significant cost to the presumed advantage of the open-ended format over the multiple-choice format in testing higher orders of cognition. Feletti and Smith have shown that as the number of items in the open-ended examination increases, questions probing high-order cognitive skills tend to be replaced by questions requiring factual recall alone [46]. Hence as accuracy and reliability increase, any difference between such an assessment and a multiple-choice assessment in terms of other indicators tends to disappear; ultimately they converge on an essentially identical assessment [47],[49].

Palmer and Devitt [45] analysed a large number of multiple-choice and MEQ questions used for summative assessment in a clinical undergraduate exam. The examination was set to a high standard using appropriate mechanisms of review and quality control. Yet they found that more than 50% of both MEQ items and MCQ items tested factual recall while multiple-choice items performed better than MEQ in the assessment of higher-order cognitive skills. They reported that "the modified essay question failed in its role of consistently assessing higher cognitive skills whereas the multiple-choice frequently tested more than mere recall of knowledge”.

In a subsequent study of a rigorously prepared and controlled set of exit examinations, they reported that the proportion of questions testing higher-level cognitive skills was lower in the MEQ paper then in the MCQ paper. More than 50% of the multiple-choice items assessed higher level cognition, as opposed to just 25% of the MEQ items. The problem was compounded by a higher frequency of item-writing flaws in the MEQ paper, and flaws were found in the marking scheme in 60% of the MEQ's. The authors conclude that “The MEQ paper failed to achieve its primary purpose of assessing higher cognitive skills” [47].

We therefore appear to be dealing with a general rule: the more highly open-ended questions are structured with the intention of increasing reliability, the more closely they converge on an equivalent multiple-choice question in terms of performance, thus negating any potential advantage of the open-ended format over the closed-ended [53]; indeed they appear frequently to underperform MCQ items in the very area in which they are believed to hold the advantage. Thus the shift to these newer forms of assessment may actually have had a perverse effect in diminishing the potential for the open-ended assessment to evaluate complex cognitive processes. This does not imply that open-ended items such as SAQ, MEQ and key-feature assessments, particularly those designed to assess clinical reasoning, are inherently inferior to MCQ; rather it is a warning that there is a very real risk in practice of “dumbing-down” such questions in an attempt to improve reliability, and empiric observations suggest that this is indeed a consequence frequently encountered even in carefully crafted assessments.

Combining multiple-choice and open-ended tests in the same assessment, in the belief that one is improving the strength of the assessment, leads to an overall less reliable assessment than is constituted by the multiple-choice section on its own [49], thus causing harm rather than adding benefit [50].

The second argument, frequently advanced in support of the open-ended format, is that it has greater validity; that spontaneously recalling and reproducing knowledge is a better predictor of the student’s eventual ability to handle complex problems in real-life then is the ability to select an answer from a list [54]. Indeed, this argument is intuitively highly appealing. The case for the retention of open-ended questions in medical undergraduate and postgraduate assessment largely rests on validity, with the assumption that *asking* the candidate to describe how they would diagnose, investigate and treat a patient predicts future clinical competence more accurately than does the ability to *select* the right response from a number of options [55],[56]. The question of validity is central. If the open-ended format is genuinely of higher validity than the multiple-choice format, then there is a strong case for retaining essay-type questions, SAQ and MEQ in the assessment protocol. If this contention cannot be supported, then the justification for retaining open-ended items in summative assessment may be questioned.

Is the contention true? Essentially, this may be explored at two levels. The first is to correlate outcomes between the two formats. The second is to perform appropriate statistical analysis to determine whether these formats are indeed testing different dimensions or “factors”.

### Validity

Validity is an indicator of how closely the assessment actually measures the quality it purportedly sets out to test. It is self-evident that proficiency in many domains, including clinical practice, requires not only the ability to recall factual knowledge, but also the ability to generate and test hypotheses, integrate knowledge and apply it appropriately as required.

Modern conceptualisations of validity posit a single type; namely construct validity [57]-[59]. This is based on the premise that ultimately all validity rests on the fidelity with which a particular assessment reflects the underlying construct, “intangible collections of abstract concepts and principles which are inferred from behaviour and explained by educational or psychological theory” [60]. Construct validity is then defined as a process of investigation in which the constructs are carefully delineated, and evidence at multiple levels is sought which supports a valid association between scores on that assessment and the candidate's proficiency in terms of that construct. For example, five types of evidence have been proposed which may provide support for such an association [60],[61], namely content, the response process, internal structure, relationship to other variables and consequences. In this discussion we highlight the relevant to the last two methods; convergent correlations between the two forms of assessment, and the impact of test scores on later performance, particularly that requiring problem-solving under conditions encountered in the work situation. This “is particularly important to those employers more interested in hiring competent workers than good test takers” [62].

### Direct comparisons of the open-ended and multiple-choice formats

#### Correlation

Numerous studies have assessed the correlation of scores between the two formats. If scores are highly correlated, the two formats are essentially measuring the same thing in which case, in terms of validity, there is no advantage of one over the other. With few exceptions, studies indicate that scores on the two forms of assessment are highly correlated. Norman *et al.* compared the two formats prospectively and showed a strong correlation between the two sets of scores [63]. A similar result was found by Palmer *et al.* who suggested that the two types of examination were essentially testing similar characteristics [47]. Similarly Norcini *et al.* found that written patient management problems and multiple choice items appeared to be measuring essentially the same aspects of clinical competence, though the multiple-choice items did so more efficiently and with greater reliability [17]. Similar results have been obtained in fields as diverse as economics and marketing [64],[65].

In general correlations between the two formats are higher when the questions in each format are specifically designed to be similar (stem-equivalent), and lower where the items in the two formats differ. However, the difference is not great: in a meta-analysis, Rodriguez found a correlation across 21 studies of 0.92 for stem-equivalent items and 0.85 across 35 studies for non-stem-equivalent items. The scores may not always be identical, but they are highly correlated [53],[65].

#### Factor analysis: do the formats measure more than one construct?

Identification of the actual constructs measured in an assessment has proved challenging given the lack of congruence between the simple cognitive assumptions on which testing is often based and the very complex cognitive nature of the constructs underlying understanding [66]. A number of studies have used confirmatory factor analysis and principal component analysis to determine whether the constructs tested by the two formats lie along a single dimension or along two or more divergent dimensions. Bennett *et al*. compared a one factor model with a two factor model to examine the relationship of the open-ended and closed-ended formats and found that in general the single factor provided a better fit. This suggests that essentially the two formats are testing the same thing [67]. Similarly Bridgeman and Rock found, using a principal components model, that both formats appeared to load on the same factor, implying that the open-ended format was not providing information on a different dimension [68]. Thissen and Wainer found that both formats could largely be ascribed to a single shared factor but did find some specific open-ended factors for which only the open-ended items contributed [69]. Though Lissitz *et al*. [70] quote a study by JJ Manhart, which found a two-factor model generally more appropriate than a one factor model, this study has not been published and the significance of the divergence cannot be assessed.

In a study of high school assessments using confirmatory factor analysis, Lissitz *et al.* showed a correlation of 0.94 between the two formats in the domains of algebra and biology; a two-factor model provided a very slight increment over a one-factor model in terms of fit. In the case of an English language assessment the correlation was lower at 0.74 and a two-factor model provided a better fit. In a test of US government, intermediate results were found with the correlation of 0.83 and a slight superiority of a two-factor model. This suggests that the addition of open-ended items in biology and algebra provided little further information beyond the multiple-choice items, whereas in other domains—English and government—the two formats are to some degree measuring different constructs [70]. Indeed, the literature in general suggests that differences in format appeared to be of little significance in the precise sciences such as biology and mathematics, but may have some relevance in fields such as history and languages, as suggested by Traub and Fisher [71]. In summary, there is little evidence to support the belief that the open-ended format is testing dimensions which the multiple-choice format cannot [53],[70],[72].

Construct validity was specifically assessed by Hee-Sun *et al*. [73], who attempted to measure the depth of understanding among school-level science students revealed by multiple-choice and short written explanatory answers respectively. They reported that students who showed higher degrees of knowledge integration were more likely to score highly on multiple-choice, though the reverse did not hold true. They suggested that the multiple-choice items were less effective in distinguishing adjacent grades of understanding as opposed to distinguishing high-performance from low performance, a finding similar to that of Wilson and Wang [74] and Ercikan *et al*. [75]. Unfortunately the generalisability of these results is limited since the multiple-choice items were poorly standardised, both in format and in difficulty, and the circumstances under which the testing was conducted were essentially uncontrolled.

Lukhele *et al*. performed a rigorous analysis of high-quality university placement exams taken by thousands of candidates [49]. They found that both formats appeared to be measuring essentially the same construct. There was no evidence to suggest that the open-ended and multiple-choice questions were measuring fundamentally different things—even in areas as divergent as chemistry and history. Factorial analysis suggested that there were two variant dimensions reflected in the scores of the multiple-choice and open-ended sections, one slightly more related to multiple-choice and the other to the open-ended format. However these were highly correlated, whatever the factor is that is specifically measured by the open-ended format, multiple-choice would measure it almost as well. Thus for all practical purposes, in such summative assessments, multiple-choice assessments can satisfactorily replace open-ended assessments.

**Figure 3:**
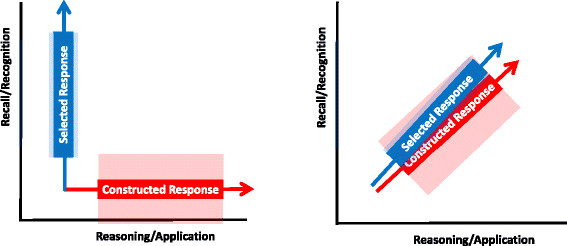
**Stylized depiction of the contrasting ability of the presumed open-ended and multiple-choice formats to assess recognition and recall as opposed to higher forms of cognitive learning.** Ideally, multiple-choice and open-ended questions would measure two different abilities (such as recall/recognition versus reasoning/application) – this may be shown as two divergent axes (shown on left). The error variance associated with each type of question is indicated by the shaded blocks, and is much greater for the open-ended question, given its inherent lower reliability. In practice, it appears that the two axes are closely aligned, implying that the two types of questions are measuring essentially the same thing (shown on right). What little additional information the open-ended question might be giving (as shown by a slight divergence in axis) is offset by its wide error variance, which in effect overlaps the information given by the multiple-choice question, thus significantly reducing the value of any additional information it provides.

An important principle is that the variance introduced by measuring “the wrong thing” in the multiple-choice is small in comparison with the error variance associated with the open-ended format given its low reliability. This effectively cancels out any slight advantage in validity [49] (Figure 3). Indeed, Wainer and Thissen state that “measuring something that is not quite right accurately may yield far better measurement than measuring the right thing poorly” [50].

In summary, where studies have suggested that the open-ended format is measuring something that multiple-choice does not (particularly in older studies), the effect has tended to be minimal, or possibly explicable on methodological grounds, or indefinable in terms of what is actually being measured. In contrast, methodologically sound studies converge on the conclusion that the difference in validity between the two formats is trivial. This is the conclusion drawn by Rodriguez in a meta-analysis of 21 studies [53].

Demonstrating an essential similarity for the two formats under the conditions of summative assessment does not necessarily mean that they provide identical information. It is possible and indeed likely that open-ended questions may make intermediate steps in thinking and understanding visible, thus serving a useful role in diagnostic as opposed to summative assessment [73],[75],[76]. Such considerations are particularly useful in using assessment to guide learning rather than merely as a judgment of competence [77]. In summative assessment at a stage prior to final exit from a programme, and particularly in formative assessment, the notion of *assessment for learning* becomes important; and considerations such as the generation effect and the potentiation of memory recall by testing cannot be ignored. Interestingly, a recent publication suggests that multiple-choice format testing is as effective as SAQ-format testing in potentiating memorisation and recall [23], thus supporting the contention that well-crafted MCQ and open-ended questions are essentially stimulating the same cognitive processes in the learner.

Some authors have raised the concern that students may constitutionally perform differentially on the two forms of assessment, and might be disadvantaged by a multiple-choice assessment should their strengths lie in the open-ended format. Studies in this area have been reassuring. Bridgeman and Morgan found that discrepant results were not predictive of poor academic performance as assessed by other parameters [78]. Ercikan *et al*. reported that discrepancies in the outcome between open-ended and multiple-choice tests were largely due to the low reliability of the open-ended component and inappropriate testing strategies [75]. A study which correlated the two formats with each other and with other measures of student aptitude showed a high degree of correlation and was unable to identify students who clearly had a propensity to perform consistently better on one format than the other [79]. Thus the belief that some students are constitutionally more suited to open-ended questions than to multiple-choice would appear to be unfounded.

An important question is whether the format of assessment effects the type of learning students use in preparation for it. As early as 1971, Hakstian suggested that anticipation of a specific form of examination did not result in any change in the amount or type of preparation, or any difference in performance in subsequent testing [80]. He concluded as follows: “The use of various types of tests to foster various kinds of study and learning, although widely advocated would seem to be a practice based on intuitive appeal, but not convincingly supported by empirical research. In particular, the contention that the superiority of the essay examination is its ability to promote more desirable study methods and higher performance on tasks requiring organisation, and deeper comprehension analysis of information should be re-evaluated in light of the evidence in the present study of no differences between groups in terms of study methods, the essay examination, or items from the higher levels of the cognitive domain”. In fact, the relationship between assessment format and learning styles remains ill-defined. Though some studies have suggested that students tended to make more use of surface learning strategies in preparation for MCQ and deeper learning strategies in preparation for open-ended questions [81],[82], other studies have failed to show such an association [80],[83]. Some studies have even failed to show that deep learning approaches correlated with better performance in applied MCQ’s and a written course project, both of which required high level cognitive performance [84],[85], though, a significant finding was that a surface learning strategy appeared deleterious for both factual and applied MCQ scores [85].

Indeed, a review of the literature on learning strategies suggests that the notion that one or other assessment format consistently calls forth a particular learning strategy is simplistic, and much of the evidence for this may have been misinterpreted [86]. The student’s choice of learning style appears to be dependent on multiple interacting and to some extent, confounding factors, most importantly the student’s innate learning motivation and preferred learning strategy. This is however subject to modification by other factors, particularly the student’s own perception of whether the assessment is directed at assessment of factual knowledge or of understanding, a perception which may frequently not coincide with the intentions of the examiner [87]. Individual differences in learning strategy probably outweigh any other consideration, including the assessment format, though this is not constant and students will adapt their preferred learning strategy according to their perception of the requirement for a particular assessment [88]. A further study has suggested that the approach to learning the student brings into the course is the strongest predictor of the learning style they will employ subsequently and, irrespective of the instructor’s best efforts, the only factor significantly correlated with the change in learning style is a change in the student’s perception of the cognitive demands of the assessment. Thus students are frequently strategic in their choice of learning strategy, but the strategies may be misplaced [87]. The student’s academic ability may be relevant; one study has shown that more academically able science students correctly identified the MCQ as requiring deep knowledge and adopted an appropriate learning strategy, whereas less able students interviewed the assessment as principally a test of recall and used a counter-productive surface-learning strategy.

Hadwin *et al*. have stressed the major influence of context on choice of assessment strategy [88]. There is for example evidence that students will modify their strategy according to whether the assessment is perceived as a final examination or as an interim assessment, irrespective of format [81]. So-called construct-irrelevant factors such as female gender and increasing maturity tend to correlate with selection of a deep learning strategy [85] independent of assessment format, while the association of anxiety and other emotional factors with a particular assessment will impair performance and thus operate as a confounding factor [89],[90]. In discussing their results, Smith and Miller stated that “Neither the hypothesis that multiple-choice examination will promote student use of surface strategy nor the hypothesis that essay examination will promote student use of deep strategy were supported” [91]. As a general conclusion, it would appear valid to say that current evidence is insufficient to suggest that the open-ended format should be preferred over MCQ or vice versa on the grounds that it promotes more effective learning strategies.

It is also important to be aware that open-ended assessments may bring confounding factors into play, for example testing language mastery or skills rather than the intended knowledge domain itself [70], and hand-written answers also penalise students with poor writing skills, low writing speeds and poor handwriting [65].

#### Prediction

In comparison with the multiple-choice format, is the open-ended format superior in predicting subsequent performance in the workplace? This has been assessed and the answer, surprisingly, is that it may be less predictive. Rabinowitz and Hojat [92] correlated the single MEQ assessment and five multiple-choice assessments written at the conclusion of a series of six clerkships with performance after graduation. Results in multiple-choice assessment consistently demonstrated the highest correlations with subsequent national examination scores and with objective assessments of performance in the workplace. The MEQ questions showed the lowest correlation. Wilkinson and Frampton directly compared an assessment based on long and short essay-type questions with a subsequent assessment protocol containing short essay questions and two multiple-choice papers [56], correlating these with performance in the subsequent internship year using robust rating methodologies. They found no significant correlation between the scores of the open-ended question protocol and assessments of performance in the workplace after graduation. In contrast they found that the combination of the SAQ paper and two multiple-choice papers showed a highly significant correlation with subsequent performance. This study showed that the predominant use of multiple-choice in the assessment resulted in a significant improvement in the structural validity of the assessment in comparison with essay-type questions alone. It was unable to answer the question as to whether the open-ended questions are necessary at all since the multiple-choice component was not compared with the performance rating independently of the essay questions. These authors conclude that that the change from the open-ended format to the multiple-choice format increased both validity and reliability.

### Recommendations from the literature

Wainer and Thissen stated that: “We have found no evidence of any comparison of the efficacy of the two formats (when a particular trait was specified and skilled item writers then constructed items to measure it) in which the multiple-choice item format was not superior” [50]. Lukhele *et al*. concluded: “Thus, while we are sympathetic to… the arguments… regarding the advantages of open-ended format, we have yet to see convincing psychometric evidence supporting them. We are awash in evidence of their drawbacks”, and further, “… We are forced to conclude that open-ended items provide this information in more time at greater cost than the multiple-choice items. This conclusion is surely discouraging to those who feel that open-ended items are more authentic and, hence, in some sense, more useful than multiple-choice items. It should be” [49].

Palmer *et al*. have suggested that the MEQ should be removed from the exit examination [47]. Given that MEQ's are difficult to write to a high standard and in such a way that they test high-order cognitive skills, and given the time required and the subjectivity in marking, their use does not represent an efficient use of resources. Indeed, they state “… MEQ's often do little more than test the candidate's ability to recall a list of facts and frustrate the examiner with a large pile of papers to be hand-marked”. They conclude there is no good measurement reason for including open-ended items in the high-stakes assessment, given that the MEQ performed poorly in terms of testing high-order thinking in comparison with the multiple-choice despite considerable effort to produce quality questions.

Schuwirth and Van der Vleuten too have suggested that there is no justification for the use of SAQ in assessment, since the stimulus of most SAQ can also be applied with multiple-choice. They recommend that SAQ should not be used in any situation except where the spontaneous generation of the answer is absolutely essential. Furthermore, they believe that there is little place for context-free questions in medical assessment as the context-rich stimulus approximates clinical practice more closely [48].

#### Why does the open-ended format persist in medical assessment?

Hence the evidence suggests that in written summative assessment the multiple-choice format is no less able to test high-order thinking than open-ended questions, may have higher validity and is superior in reliability and cost-effectiveness. Remarkably this evidence extends as far back as 1926 [53],[93], and the reasons underlying the persistence of the open-ended format in assessment are of some interest. I suggest a number of factors. Studies bear out the common-sense expectation that questions designed to test factual knowledge only—irrespective of whether these are presented as open-ended or in multiple-choice format—do not test the same level of reasoning as more complex questions [94]. Indeed, a recurring finding in the literature is that the so-called deficiencies of the multiple-choice format lie more with the quality of the individual question item (and by inference, with the question-setter), than with the format *per se*. This leads to a self-fulfilling prophecy: examiners who do not appreciate the versatility of the multiple-choice format set questions which only test low-order thinking and not surprisingly achieve results which confirm their bias. Palmer *et al.* state that criticism of multiple-choice as being incapable of testing high-order thinking is in fact criticism of poorly written questions, and that the same criticism can be directed at open-ended assessments [45]. There is indeed evidence that stem-equivalent items tend to behave similarly, irrespective of whether the item is phrased as an open-ended question or in MCQ format. It is therefore essential that in making comparisons, the items compared are specifically crafted to assess the same order of cognition. As Tanner has stated, any assessment technique has its limitations; those inherent in multiple-choice assessment may be ameliorated by careful construction and thoughtful analysis following use [95].

Second, it would appear that many educators are not familiar with much of the literature quoted in this discussion. The most persuasive material is found in the broader educational literature, and though there are brief references in the medical education literature to some of the studies to which I have referred [47],[48], as well as a few original studies performed in the medical assessment context [17],[45],[47],[63], the issue does not appear to have enjoyed prominence in debate and has had limited impact on actual assessment practice. In their consensus statement and recommendations on research and assessment, Schuwirth *et al.* stress the need for reference beyond the existing medical education literature to relevant scientific disciplines, including cognitive psychology [27]. In the teaching context, it is remarkable how the proposition that the open-ended format is more appropriate in testing the knowledge and skills ultimately required for the workplace has been repeatedly and uncritically restated in the literature in the absence of compelling evidence to support it.

Third is the counter-intuitiveness of this finding. Indeed, the proposition that the open-ended format is more challenging than MCQ is intuitively appealing. Furthermore, there is the “generation effect”; experimental work has shown that spontaneous generation of information, as opposed to reading enhances recall [18],[19]. Although this applies to learning rather than to assessment, many teachers implicitly attribute a similar but reversed process to the act of recall, believing that spontaneous recall is more valid than cued recall. However, validity at face value is an unreliable proxy for true validity, and the outcome in practice may contradict what seems intuitively correct [48]. As the literature on learning increases, it has become apparent that evidenced-based practice frequently fails to coincide with the intuitive appeal of a particular learning methodology. Examples include the observation that interleaved practice is more effective than blocked practice and distributed practice is more effective than massed practice in promoting acquisition of skills and knowledge [21]. There is a need for assessment to be evidence-based; to an extent assessment would appear to lag behind learning and teaching methodology in this respect. Rohrer and Pashler have suggested that underutilisation of learning strategies shown to be more effective than their traditional counterparts, such as learning through testing, distributed practice and interleaved practice, remain so because of “the widespread (but erroneous) feeling that these strategies are less effective than their alternatives” [21].

Fourth and perhaps most defensible is concern that there is much that as yet remains unknown about the nature of assessment; particularly seen from the viewpoint of *assessment for learning,* and given very interesting new insights into the cognitive basis of memorisation, recall and reasoning, a field which is as yet largely unexplored, and may be expected to have a significant impact on the choice of assessment format. For diagnostic purposes, the open-ended format may hold value, since it is better able to expose the students intermediate thinking processes and therefore allow precise identification of learning difficulties [72]. Newer observations such as the generation effect [18],[19], the testing effect [20],[23], the preassessment effect, where the act of preparation for an assessment is itself a powerful driver of learning [96], and the post-assessment effect, such as the effect of feedback [96] are clearly important; were it to be shown that a particular format of assessment, such as the open-ended question, was superior in driving learning, then this would be important information which might well determine the choice of assessment. At this point however no such reliable information exists. Preliminary work suggests that MCQ items are as effective as open-ended items in promoting the testing effect [23]. None of these considerations are as yet sufficiently well supported by experimental evidence to argue definitively for the inclusion of open-ended questions on the basis of their effect on learning, though the possibility clearly remains. Furthermore, this debate has concentrated on high-stakes, summative exit assessments where the learning effects of assessment are presumably less important than they are at other stages of learning. Certainly, open-ended assessment remains appropriate for those domains not well-suited to multiple-choice assessment such as data gathering, clinical judgement and professional attitudes [92] and may have value for a particular question which cannot be presented in any other format [48]. Though the evidence is less compelling, open-ended items may be superior in distinguishing between performances of candidates occupying the two extremes of performance [75].

### Cognitive basis for the observation

The need for assessment of research to move beyond empiric observations to studies based on a sound theoretical framework has recently been stressed [27],[96]. There is as yet little written on the reasons for the counter-intuitive finding that MCQ is as valid as open-ended assessments in predicting clinical performance. I suggest that the observation is highly compatible with cognitive-constructivist and situated learning theory, and in particular the theory of conceptual change [97]. Fundamental to this theory is the concept of mental models. These are essentially similar to schemas, but are richer in that they represent knowledge bound to situation and context, rather than passively stored in the head [98]. Mental models may therefore be thought of as cognitive artifacts constructed by an individual based on his or her preconceptions, cognitive skills, linguistic comprehension, and perception of the problem, which evolve as they are modified through experience and instruction [99]. Conceptual change is postulated to represent the mechanism underlying meaningful learning, and is a process of progressively constructing and organizing a learner’s personal mental models [100],[101]. It is suggested that an effective mental model will integrate six different aspects: knowledge appropriately structured for a particular domain (structural knowledge), pathways for solving problems related to the domain (procedural knowledge), mental images of the system, associations (metaphors), the ability to know when to activate mental models (executive knowledge), and assumptions about the problem (beliefs) [102]. Therefore increasing proficiency in any domain is associated not just with an enlarging of store of knowledge and experience, but also with increasing complexity in the extent to which knowledge is organised and the manner in which it is stored and accessed [103], particularly as complex mental models which may be applied to problem-solving [104]. A counterpart in the domain of medical expertise is the hierarchy of constructs proposed by Schmidt *et al*. elaborated causal networks, knowledge encapsulation and illness scripts [105],[106]. Conceptual change theory has a clear relationship to our current understanding of expertise, which is postulated to emerge where knowledge and concepts are linked as mental representations into propositional networks which allow rapid processing of information and the omission of intermediate steps in reasoning [107],[108]; typically the expert’s knowledge is grouped into discrete packets or chunks, and manipulation of these equates to the manipulation of a large amount of information simultaneously without conscious attention to any individual component [104]. In comparison with non-experts, the representations of experts are richer, more organised and abstract and are based on deep knowledge; experts also recognise the conditions under which use of particular knowledge is appropriate [109]. As Norman has stated, *“expert problem-solving in medicine is dependent on (1) prior experiences which can be used in routine solution of problems by pattern recognition processes and (2) elaborated conceptual knowledge applicable to the occasional problematic situation*” [110]. The processes of building expertise and that of constructing mental models are essentially parallel [99].

Therefore any form of assessment intended to measure proficiency must successfully sample the candidate’s organisation of and access to knowledge, and not just content knowledge alone [99],[111]. I have reviewed the empirical evidence which suggests that the multiple-choice format is indeed predictive of proficiency, which provides important evidence that it is valid. This is explicable in terms of mental models. An alternative view of a mental model is as *an internal representation of a system that the learner brings to bear in a problem-solving* situation [103],[104],[112]. The context-rich written assessment [48] is essentially an exercise in complex problem-solving, and fits the definition of problem-solving as “cognitive processing aimed at accomplishing certain goals when the solution is unknown” [103],[113].

Zhang has introduced the concept of a “distributed cognitive task”: a task requiring that information distributed across both the internal mind and the external environment is processed [114]. If we extend Zhang’s concept of external representation to include a hypothetical patient, the subject of the clinical vignette, who represents the class of all such patients, then answering the context-rich multiple-choice item may be seen as a distributed cognitive task. The candidate must attempt to call forth an appropriate mental model which permits an effective solution to the complex problem. In a sequence of events which parallels that described by Zhang, the candidate must internalise the information provided in the vignette, form an accurate internal representation (an equivalent concept is that of the problem space, a mental representation of the problem requiring solution [115]); this in turn activates and interacts with the relevant mental models and is followed by externalization: the return of the product of the interaction of internal representation and mental model to the external environment, and the selection of a solution. In effect a relationship has been defined between environmental information, activation of higher level cognition and externalisation of internal representations [114].

Assessment items which require complex problem-solving call on mental models appropriate to that particular context, and the item can only be answered confidently and correctly if the mental model is present at the level of proficiency. There is therefore no such thing as the student with generic expertise “in answering multiple-choice questions”, which explains the findings of Hakstian [80], Bridgeman and Morgan [78], Ercikan *et al.* [75] and Bleske-Rechek *et al*. [79], none of whom found convincing evidence for the existence of a class of student with a particular skill in answering multiple-choice questions.

Recent observations that retrieval of knowledge improves retention, and may be enhanced in the learning process by frequent testing [20],[21], and in particular a recent publication summarising four studies performed in an authentic learning environment which demonstrates that that testing using MCQ format is as effective as SAQ testing [23], supports the hypothesis that the MCQ format engages with high order cognitive processes, in both learning and retrieval of memory. This is further supported by their finding that high-level test questions stimulate deeper conceptual learning and better learning retention then do low-level test questions [23].

In summary, the multiple-choice item is testing the integrity and appropriateness of the candidate’s mental models, and in doing so, is in fact assessing proficiency. If the item is designed to test factual recall only then it will fail for this purpose, since it is the solution of a complex problem which tests the strength of the mental model and the cognitive processes which interact with it. Yet even a low-quality assessment based on factual recollection will correlate significantly with proficiency. Firstly, all mental models are based on a foundation of structural knowledge. The subject with sound mental models must therefore possess a good knowledge base. Secondly, possessing effective and appropriate mental models facilitates the retention and recall of knowledge [103]. Not surprisingly therefore, even on a fact-based assessment, good students will correctly recall the information and excel; students with deficient mental models, are less likely to be able to recall the information when needed. This is supported by the work of Jensen *et al*. [116] who found that high order questions stimulated deep conceptual understanding and retention, and correlated with higher performance on both subsequent high order assessment items and low-order assessment items. Indeed, recognition and recall are highly correlated [50]. There is evidence that the cognitive processes evoked by the multiple-choice format are not influenced by cueing [117], though the reasons for the frequent observation that MCQ scores are higher than those for equivalent open-ended item assessments raise concern that cueing may yet have a role [118]. However, where the stem and options have been well-designed―particularly such that the distractors all appear attractive to the candidate without the requisite knowledge― cueing should not be an issue [29],[48], and the common argument that it is easier to recognize an answer than it is to generate it spontaneously would appear not to hold true.

Problem-solving skills are poorly generalizable [41]. This is explicable in that mental models are essentially domain-specific, representing a particular set of knowledge and circumstances, but the actual process of developing them is highly dependent on domain-general processes including metacognition, self-regulation and cognitive flexibility [99].

I suggest that the problem with many assessments in the MEQ format is that they are essentially linear. By requiring the candidate to think one step at a time, the assessment effectively misses the crux of the problem-solving process, which is to look at and respond to a complex problem in its entirety, and not stepwise. The context-rich vignette-based multiple-choice item by contrast presents a complex problem which must be holistically assessed. Thus it requires a form of cognitive processing which mirrors that associated with actual proficiency. Hybrid formats such as key feature assessments in effect also break down the clinical reasoning process into a sequence of sequential steps; whether this is regarded as a drawback will depend on the relative importance ascribed to decision-making at critical points in the decision tree and global assessment of a problem viewed holistically. This is a critical area for future research in clinical reasoning.

Educators who mistrust the multiple-choice format have tended to concentrate on the final, and cognitively the least important, step in this whole process: the selection of a particular option as the answer, while ignoring the complex cognitive processes which precede the selection. Indeed, in a good assessment, the candidate is not “selecting” an answer at all. They recognise the external representation of a problem, subject the internalised representation to high level cognitive processing, and then externalise the product as a solution [119], which (almost as if coincidentally) should coincide with one of the options given.

The multiple-choice format is by no means unlimited in its capacity to test higher-order thinking. The literature on problem-solving stresses the importance of highly-structured complex problems, characterised by unknown elements with no clear path to the solution and indeed a potential for there to be many solutions or even no solution at all [99]. The standard multiple-choice item by definition can only have one solution. Thus, though it may be context-rich, it is limited in its complexity. It is difficult however to imagine how a practically achievable open-ended written assessment might perform better. In order to accommodate complexity, the question would essentially have to be unstructured—thereby eliminating all the structured short-answer progeny of the essay format, such as MEQ. In order to permit the candidate to freely demonstrate the application of all his or her mental resources to a problem more complex than that permitted by a multiple-choice vignette, one would in all probability require that the candidate is afforded the opportunity to develop an extensive, unstructured and essentially free-ranging, essay-length response; marking will be inherently subjective and we are again faced with the problem of narrow sampling, subjectivity and low reliability.

In effect the choice would then lie between an assessment comprising one or two unstructured essay length answers with low objectivity and reliability, and a large number of highly reliable multiple choice items which will effectively test high-order problem-solving, but will stop short of a fully complex situation. Perhaps this is a restatement of the assertion that “measuring something that is not quite right accurately may yield far better measurement than measuring the right thing poorly” [50], the situation depicted in Figure 3.

Another way of understanding the validity of the multiple-choice format is by comparing the responses of candidates at different phases of the learning process with the stages of increasing proficiency posited by Dreyfus *et al*. [16] (Table 1). Here the first column comprises the stages of learning; in this context, we shall regard stage of learning as synonymous with level of proficiency or expertise, which is a measure of the effectiveness of problem-solving skill. The second column contains descriptors for each stage chosen for their relevance to complex problem-solving posed by a well-constructed context-rich multiple-choice item. The third column contains a description of the likely performance on that item of a candidate at that stage of proficiency. The relationship between proficiency and performance in a complex multiple-choice item is in fact remarkably direct. The candidate who has reached the stage of proficiency or expertise will be more likely to select the correct response than candidates at a lower level, and the more widely such proficiency is spread across the domain, the higher the aggregate score in the assessment. Though the score for a standard multiple-choice item is binary (all or nothing), the assessment as a whole is not. Whereas candidates in the top categories are likely to arrive at a correct solution most of the time, and students in the lowest category hardly ever, the middle order candidates with less secure mental models will answer with less confidence, but will in a number of items proportional to their proficiency, come up with the correct solution, their mental models proving to be sufficiently adequate for the purpose. Over a large number of items such a multiple-choice assessment will therefore provide a highly accurate indication of the level of proficiency of the candidate. To avoid all confounding variables however it is absolutely essential that the options are set such that cueing is eliminated.

**Table 1:** **Adapted and extended from Kim** [100]

Stage	Description	Expected performance ***in a well-structured context-rich multiple-choice assessment requiring complex problem-solving***
Novice	Knowledge lacks structure and is essentially context-free. Concepts and relationships are of poor quality	The candidate will be unable to identify or contextualise the problem. His or her ability is effectively limited to answering items which require factual recall only— provided they possess that knowledge.
Advanced beginner	Situated knowledge is present but cannot be prioritised appropriately for the problem	Though the problem may be recognised, the candidate will be unable to represent it internally or activate a mental model with sufficient fidelity for problem-solving.
Competent learner	Is able to extract the key elements from the problem and possesses many or most of the concepts required for application to the problem, but the relationship between these may not yet be fully mature.	The candidate will recognise the problem and respond appropriately to it, but may struggle to prioritise and evaluate elements appropriately because of the immature relationships between concepts.
Proficient learner	Immediately recognises the problem and is able to accommodate it fully in a mental model which permits a solution.	Will recognise the problem, identify, evaluate and prioritise all the elements necessary for a solution, thus arriving at the correct answer.
Intuitive expert	Understands and responds to the situation intuitively, using tacit knowledge arising from extensive experience	Is able to answer the question intuitively with minimal analysis or thinking.

The first column comprises the stages of learning proposed by Dreyfus and Dreyfus [16]; in this context, we shall regard stage of learning as synonymous with level of proficiency or expertise, which is a measure of the effectiveness of problem-solving skill. The second column contains descriptors for each stage chosen for their relevance to complex problem-solving posed by a well-constructed context-rich multiple-choice item. The third column contains a description of the likely performance on that item of a candidate at that stage of proficiency. The relationship between proficiency and performance in a complex multiple-choice item is in fact remarkably direct.

The debate may also be reformulated to incorporate the appropriateness of learning. Deep learning is characterised by an understanding of the meaning underlying knowledge, reflection on the interrelationships of items of information, understanding of the application of knowledge to everyday experience, integration of information with prior learning, the ability to differentiate between principle and example and the organisation of knowledge into a coherent, synthetic structure [99],[100]—essentially an alternative formulation of the mental model. One can thus argue that the candidate who possesses deep knowledge has, by the very fact of that possession, demonstrated that they have the sort of comprehensive and intuitive understanding of the subject—in short, the appropriate mental models as described by Jonassen and Strobel [97],[101]—to allow the information to be used for problem-solving. Correspondingly, the weak student lacks deep knowledge, and this will be exposed by a well-constructed multiple-choice assessment, provided that the items are written in a manner which explores the higher cognitive levels of learning.

Therefore, if candidates demonstrate evidence of extensive, deeply-learned knowledge, and the ability to solve complex problems, be it through the medium of multiple-choice assessment or any other form of assessment, then it is safe to assume that they will be able to apply this knowledge in practice. This accounts for the extensive correlation noted between multiple-choice performance, performance in open-ended assessments, and tests of subsequent performance in an authentic environment.

## Summary

The argument that open-ended questions do not test higher order cognitive skills, and consequently lack validity, is not supported by the evidence. Some studies may have been confounded by the unfair comparison of high-order items in one format with low-order items in another. This cannot be discounted as partly responsible for the discrepancies noted in some of the work I have referenced, such as that of Hee-Sun *et al*. [73], yet where the cognitive order of the items have been carefully matched, a number of careful studies suggest that, particularly in science and medicine, the two modalities assess constructs which though probably not identical, overlap to the extent that using both forms of assessment is redundant. Given the advantage of the multiple-choice format in reliability, efficiency and cost-effectiveness, the suggestion that open-ended items may be replaced entirely with multiple-choice items in summative assessment is one which deserves careful consideration. This counter-intuitive finding highlights our lack of understanding of the cognitive processes underlying both clinical competence and its assessment, and suggests that much further work remains to be done. Despite the MCQ format’s long pedigree, it is clear that we understand little about the cognitive architecture invoked by this form of assessment. The need for a greater role for theoretical models in assessment research has been stressed [27],[96]. As illustrated in this debate, medical teaching and assessment must be based on a solid theoretical framework, underpinned by reliable evidence. Hard evidence combined with a plausible theoretical model - which must attempt to explain the observations on the basis of cognition - will provide the strongest basis for the identification of effective learning and assessment methodologies.

That the multiple-choice format demonstrates high validity is due in part to the observation that well-constructed, context-rich multiple-choice questions are fully capable of assessing higher orders of cognition, and that they call forth cognitive problem-solving processes which exactly mirror those required in practice. On a theoretical basis it is even conceivable that the multiple-choice format will show superior performance in assessing proficiency in contrast with some versions of the open-ended format; there is indeed empirical evidence to support this in practice [56],[92]. Paradoxically, the open-ended format may demonstrate lower validity than well-written multiple-choice items; since attempts to improve reliability and reduce objectivity by writing highly focused questions marked against standardised, prescriptive marking templates frequently “trivialize” the question, resulting in some increase in reproducibility at the expense of a significant loss of validity [120]. Indeed, I have argued that, based on an understanding of human cognition and problem-solving proficiency, context-rich multiple-choice assessments may be superior in assessing the very characteristics which the proponents of the open-ended format claim as a strength of that format.

Though current evidence supports the notion that in summative assessment open-ended items may well be redundant, this conclusion should not be uncritically extrapolated to situations where *assessment for learning* is important, such as in formative assessment and in summative assessment at early and intermediate stages of the medical programme given that conclusive evidence with respect to the learning effects of the two formats is as yet awaited.

## Author’s contribution

The author was solely responsible the literature and writing the article.

## Author’s information

RJH is currently Dean and Head of the School of Clinical Medicine at the University of KwaZulu-Natal, Durban, South Africa. He studied at the University of Cape Town, specialising in Internal Medicine and subsequently hepatology, before moving to Durban as Professor of Medicine. He has a longstanding interest in medical education, and specifically in the cognitive aspects of clinical reasoning, an area in which he is currently supervising a number of research initiatives.

## Authors’ original submitted files for images

Below are the links to the authors’ original submitted files for images. Authors’ original file for figure 1 Authors’ original file for figure 2 Authors’ original file for figure 3

## Abbreviations

MEQ: Modified essay question
MCQ: Multiple-choice question
SAQ: Short answer question
OSCE: Objective structured clinical examination
