# Supplementary figures and images for: Should essays and other “open-ended”-type questions retain a place in written summative assessment in clinical medicine?
Source: BMC Med Educ. 2014 Nov 28;14:249. doi: 10.1186/s12909-014-0249-2 (PMC4275935; doi:10.1186/s12909-014-0249-2)

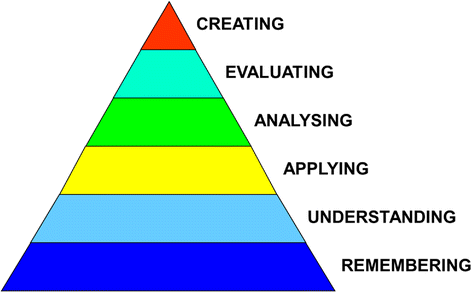

Supplement: Supplementary file 1 — Authors’ original file for figure 1 [file 12909_2014_249_MOESM1_ESM.gif]

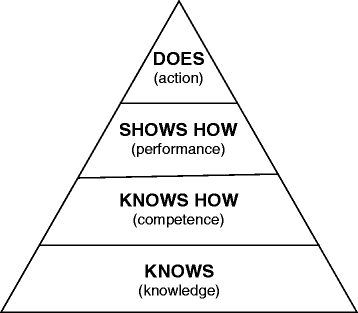

Supplement: Supplementary file 2 — Authors’ original file for figure 2 [file 12909_2014_249_MOESM2_ESM.gif]

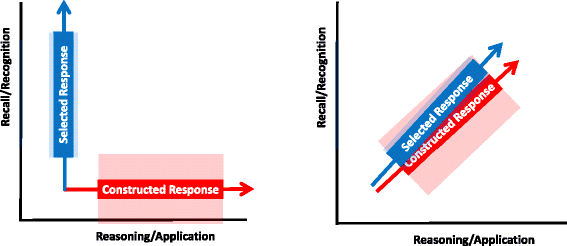

Supplement: Supplementary file 3 — Authors’ original file for figure 3 [file 12909_2014_249_MOESM3_ESM.gif]
